# Prevalence and Determinants of Undiagnosed Liver Steatosis and Fibrosis in a Nationally Representative Sample of US Adults

**DOI:** 10.7759/cureus.46783

**Published:** 2023-10-10

**Authors:** Mehul Bhattacharyya, Sharon M Nickols-Richardson, Anna L Miller, Ruemon Bhattacharyya, Frederick Frankhauser, Larry E Miller

**Affiliations:** 1 Clinical Research, Miller Scientific, Johnson City, USA; 2 Food Science & Human Nutrition, Division of Nutritional Sciences, College of Agricultural, Consumer & Environmental Sciences, University of Illinois, Urbana-Champaign, Urbana, USA; 3 Public Affairs and Economics, University of California Los Angeles, Los Angeles, USA; 4 Pharmaceutical Business & Administrative Sciences, Massachusetts College of Pharmacy and Health Sciences, Boston, USA

**Keywords:** steatosis, liver stiffness, liver disease, fibrosis, body mass index

## Abstract

Background

Chronic liver diseases account for approximately 1.9 million deaths globally every year and negatively affect health-related quality of life. Early detection of liver disease may enable timely treatment, potentially improving patient outcomes. This study aimed to determine the prevalence and determinants of liver steatosis and fibrosis in US adults with no previously diagnosed liver condition.

Methods

We conducted an observational, nationally representative, cross-sectional study using data from the National Health and Nutrition Examination Survey (NHANES) conducted from January 2017 to March 2020. Study participants were 7,391 adults aged 21 and older with no history of diagnosed liver disorders who underwent vibration-controlled transient elastography (VCTE) to determine liver steatosis and fibrosis. Controlled attenuation parameter (CAP) values between 248 and 267 dB/m were classified as mild steatosis, and those over 267 dB/m as advanced steatosis. Liver stiffness measurement (LSM) values between 7.65 and 13 kPa were classified as moderate/severe fibrosis, and those over 13 kPa as cirrhosis. Covariates included age, sex, race, body mass index (BMI), diabetes mellitus, kidney disease, smoking history, alcohol intake, alanine aminotransferase (ALT), aspartate aminotransferase (AST), physical activity, sedentary time, and sleep time. The associations of subject characteristics with liver CAP and LSM were evaluated using survey multivariable linear regression. Shapley Additive Explanations values determined the relative importance of each attribute in the model. The discriminative performance of classification models was assessed using the area under the receiver operating characteristic (AUROC) curve.

Results

The population prevalence of liver steatosis was 57.2% (10.2% mild; 47.0% advanced). The relative importance of covariates in predicting liver CAP was 63.1% for BMI, 10.7% for ALT, and less than 10% for the other covariates. The prevalence of significant fibrosis was 11.4% (8.3% moderate/severe fibrosis; 3.1% cirrhosis). The relative importance of covariates in predicting LSM was 67.3% for BMI and less than 10% for the other covariates. BMI alone demonstrated acceptable discriminative performance in classifying varying severities of steatosis and fibrosis (AUROC range 72%-78%) at cutoffs between 28 and 33 kg/m^2^.

Conclusions

Undiagnosed chronic liver disease based on VCTE findings is highly prevalent among US adults, particularly in obese individuals. Efforts to increase awareness about liver disease and to reconsider existing BMI thresholds for liver disease screening may be warranted.

## Introduction

Chronic liver diseases account for approximately 1.9 million deaths globally every year [1] and negatively affect health-related quality of life [2]. Nonalcoholic fatty liver disease (NAFLD) is present in 25% of the global population [3] and the prevalence has increased rapidly over the past decade, partly due to the increasing prevalence of metabolic syndrome [4]. The term NAFLD was traditionally defined as steatosis in more than 5% of hepatocytes in the absence of heavy alcohol consumption or known liver disease [5]. However, new terminology highlighting the link with metabolic dysfunction has been proposed [6]. Over time, liver steatosis may progress to fibrosis, cirrhosis, or hepatocellular carcinoma, with the severity of steatosis being a critical determinant in the risk of liver-related complications [7]. Currently, NAFLD and, to a lesser extent, alcoholic liver disease are the leading causes of liver transplant [8]. Early identification of individuals at risk for liver disease may enable timely intervention and improve clinical outcomes.

While liver biopsy is commonly used to qualitatively diagnose and stage liver fibrosis, procedural morbidity and risk of disease misclassification have prompted the development of non-invasive modalities for staging liver disease. Vibration-controlled transient elastography (VCTE) is an ultrasound-based test that generates a shear wave across the liver using a transducer probe. A controlled attenuation parameter (CAP) measurement is obtained from ultrasonic attenuation of the echo wave due to fat accumulation, an indicator of liver steatosis. The shear wave propagation velocity is measured and converted to a liver stiffness measurement (LSM), an indicator of fibrosis or cirrhosis. Due to the noninvasive nature of the test, VCTE is preferred over liver biopsy by 95% of patients [9] and exhibits acceptable sensitivity and specificity in detecting biopsy-confirmed advanced fibrosis [10,11]. Thus, VCTE has been increasingly utilized in liver disease screening in the general population. Vibration-controlled transient elastography was first incorporated into the National Health and Nutrition Examination Survey (NHANES) testing protocol in the 2017-2018 cycle. In the current study, we report VCTE data from NHANES between January 2017 and March 2020 to determine the prevalence and determinants of liver steatosis and fibrosis in US adults aged 21 years and older without a history of liver disease.

## Materials and methods

Source population

This cross-sectional observational study utilized data from NHANES, a continuous sample survey conducted every two years by the National Center for Health Statistics. The NHANES project is a complex, stratified, multi-stage probabilistic health survey representative of the noninstitutionalized civilian US population. The NHANES data are obtained from interviews, laboratory tests, and physical examinations conducted by trained staff. For this study, we used NHANES data from January 2017 to March 2020. The NHANES program suspended field operations in March 2020 due to COVID-19. Thus, the 2019 to March 2020 data were combined with the 2017-2018 data to create a nationally representative pre-pandemic sample. Eligible subjects for this study were US adults aged 21 years and older without a history of liver disease who underwent VCTE. The data utilized in this study were publicly available from the National Center for Health Statistics [12]. Participants provided written informed consent. The National Center for Health Statistics Ethics Review Committee granted ethics approval (Protocols #2011-17 and #2018-01). Secondary analyses of these publicly accessible data were exempt from Institutional Review Board review. This study followed the Strengthening the Reporting of Observational Studies in Epidemiology (STROBE) reporting guidelines [13].

Vibration-controlled transient elastography measurement

Trained technicians obtained a minimum of 10 VCTE measurements from fasted participants, generating median CAP (measured in decibels per meter) and LSM (measured in kilopascals) values for each participant. The CAP and LSM data were extracted from the Liver ultrasound files available from the NHANES database [12]. Higher CAP values indicate higher liver fat content, and higher LSM values indicate higher fibrosis severity. Cut-offs were derived from previous meta-analyses where CAP values between 248 and 267 dB/m were classified as mild steatosis and over 267 dB/m as advanced steatosis [14]. LSM values between 7.65 and 13 kPa were classified as moderate/severe fibrosis and over 13 kPa as cirrhosis [15].

Covariates

Covariates in this study were included based on their known clinical associations with liver disease and their feasibility of use during routine healthcare screening. Covariate data were extracted from the Alcohol use, Biochemistry, Body measures, Cigarette use, Demographic, Diabetes, Kidney conditions, Medical conditions, Physical activity, and Sleep files available from the NHANES database [12].

Demographic variables included age, sex, and race. Analyses were restricted to individuals aged 21 years and older to minimize possible reporting bias related to alcohol consumption in younger individuals. Body mass index (BMI) was calculated as weight(kg) divided by height (m^2^). Diabetes mellitus was defined as a self-reported physician diagnosis or current use of diabetes medication. Smoking history was categorized as current (smoked >100 cigarettes ever and currently smoke on some days or every day), former (smoked >100 cigarettes ever and do not currently smoke), or never (smoked <100 cigarettes ever). Alcohol intake was calculated from self-reported drinking frequency and the number of drinks consumed when drinking over the past 12 months. Heavy alcohol intake was defined as at least 15 drinks per week for men and at least eight drinks per week for women. Kidney disease was defined as a self-reported physician diagnosis of weak or failing kidneys. The serum concentration of alanine aminotransferase (ALT) and aspartate aminotransferase (AST) were included, both measured in U/L.

Physical activity was quantified as the product of work and exercise intensity by duration during a typical week, reported as the metabolic equivalent of task (MET)-minutes per week. One MET indicates the amount of oxygen consumed while sitting at rest, equivalent to 3.5 ml O_2_ per kg body weight per minute [16]. Vigorous work and leisure time activity were rated as eight METs, and moderate work activity, moderate leisure time activity, and walking or bicycling for transportation were rated as four METs [17]. Sedentary time was determined by self-reported duration of sitting during a typical day. Sleep time was determined by self-reported hours of sleep per night, averaged across weekdays and weekends.

Statistical analyses

Statistical analyses utilized complex sample methodologies incorporating sampling weights, sample strata, and clusters. The sample weight for each respondent indicated the number of individuals in the target population they represent. The sampling weights were calculated as the inverse of the selection probability, with adjustments for nonresponse patterns. Variance estimation utilized Taylor series linearization to accommodate the stratified cluster sampling design.

We compared participant characteristics across liver steatosis and liver fibrosis classifications using analysis of variance for continuous variables and chi-square tests for categorical variables. We developed multivariable linear regression models to identify determinants of liver CAP and LSM, respectively. Since identifying trivial statistically significant associations is common in population-based studies, statistical machine learning has been increasingly utilized to improve liver disease prediction models [18]. Therefore, we used Shapley Additive Explanations (SHAP) to determine the relative importance of variables in the linear regression model [19]. This game theory-based machine learning technique decomposes the model output by calculating SHAP values, ranging from 0% to 100%, which signify the relative contribution of each variable to the final model. The discriminative performance of classification models was evaluated using the area under the receiver operating characteristic (AUROC) curve, with cutoff scores determined using the Kolmogorov-Smirnov statistic [20]. All reported P-values were two-tailed and P<0.05 was considered statistically significant. Statistical analyses were conducted using Stata 18 (Released 2023; StataCorp LLC, College Station, USA).

## Results

A total of 15,560 participants were recruited into the study during the combined January 2017 to March 2020 NHANES cycle. After sequentially excluding 6,450 participants under 21 years, 480 with a history of a liver disorder (fatty liver, liver fibrosis or cirrhosis, viral or autoimmune hepatitis, liver cancer, or other liver disease), and 1,239 without VCTE data, 7,391 individuals remained in the analysis sample. These results represented approximately 211.3 million US adults aged 21 and older without a history of diagnosed liver conditions. The mean age was 48.4 years, 47.7% were male, most (63.0%) self-identified as White, mean BMI was 30.4 kg/m^2^, diabetes mellitus was reported in 9.2%, and heavy alcohol intake was reported in 6.6% of participants.

**Figure 1:**
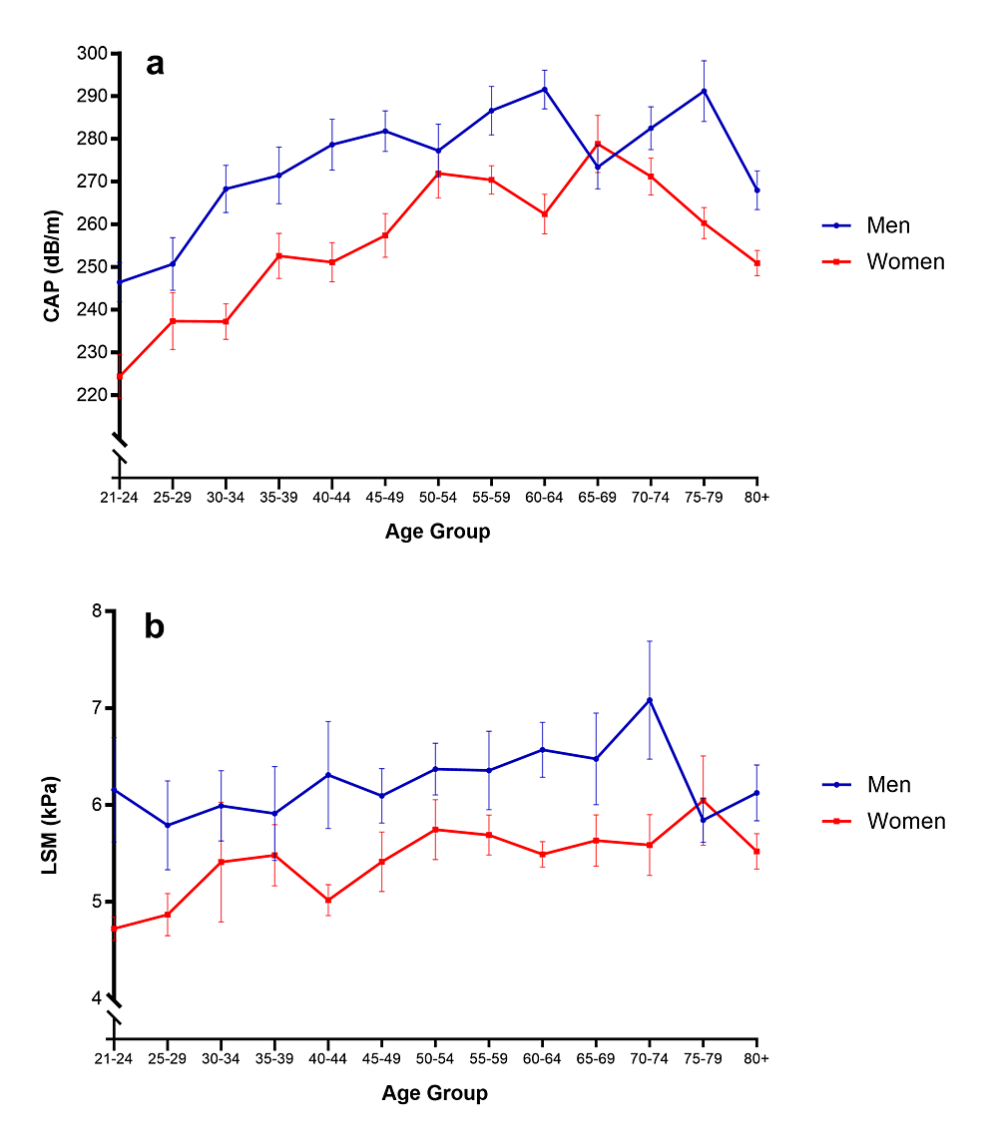
Normative values for a) CAP and b) LSM from vibration-controlled transient elastography in US adults by the age group and sex Plotted values are mean and standard error. CAP: Controlled attenuation parameter; LSM: liver stiffness measurement.

Normative population-based CAP and LSM values by age group and sex are provided in Figure 1, where values increased in both sexes until the 50s and were variable thereafter.

Overall, the prevalence of liver steatosis and/or significant fibrosis was 59.0%. The inter-relationships between VCTE-determined liver steatosis and fibrosis are displayed in Table 1.

**Table 1:** Inter-relationships of liver steatosis and fibrosis determined by VCTE results in US adults* *Values in the table represent an approximate weighted population count and percentage of adults aged 21 years and older with no history of diagnosed liver conditions. US: United States; VCTE: vibration-controlled transient elastography.

		Liver Steatosis			
		None	Mild	Severe	TOTAL
Liver Fibrosis	No/mild fibrosis	86,514,000	20,037,000	80,626,000	187,178,000
		41.0%	9.5%	38.2%	88.6%
	Moderate/severe fibrosis	2,973,000	1,463,000	13,027,000	17,463,000
		1.4%	0.7%	6.2%	8.3%
	Cirrhosis	902,000	75,000	5,644,000	6,621,000
		0.4%	0.0%	2.7%	3.1%
	TOTAL	90,390,000	21,575,000	99,297,000	211,262,000
		42.8%	10.2%	47.0%	100.0%

The population prevalence of liver steatosis was 57.2% (10.2% mild; 47.0% advanced). Comparing individuals with no, mild, or advanced liver steatosis, statistically significant differences were noted among all measured covariates (Table 2).

**Table 2:** Association of subject characteristics with liver steatosis classification assessed by VCTE* *Values are mean ± standard error or percent ± standard error unless otherwise specified. **Population of adults aged 21 years and older with no history of diagnosed liver conditions. ***Defined as ≥15 drinks per week in men and ≥8 drinks per week in women. †p<.05 considered statistically significant. ALT: Alanine aminotransferase; AST: aspartate aminotransferase; CAP: controlled attenuation parameter; MET: metabolic equivalent of task; VCTE: vibration-controlled transient elastography.

Variable	Normal (CAP < 248)	Mild Steatosis (CAP 248-267)	Advanced Steatosis (CAP ≥268)	P-value†
Population count**	90,400,000	21,600,000	99,300,000	
Population percentage**	42.8%	10.2%	47.0%	
Age, yr	44.9±0.7	49.9±0.9	51.1±0.6	<0.001
Male sex	43.0±1.3%	46.8±3.3%	54.7±1.1%	<0.001
Race				<0.001
White	63.3±2.9%	60.4±3.4%	63.3±2.6%	
Black	13.8±1.7%	11.5±1.9%	9.6±1.3%	
Asian	6.3±1.0%	6.8±1.4%	5.5±1.0%	
Mexican American	5.9±1.0%	6.8±1.0%	10.4±1.5%	
Other Hispanic	7.2±0.9%	9.5±1.4%	7.0±1.7%	
Other race / multi-race	3.5±0.5%	5.0±1.0%	4.3±0.6%	
Body mass index (kg/m^2^)	25.8±0.2	29.2±0.4	33.4±0.2	<0.001
Diabetes mellitus	3.8±0.4%	9.1±1.1%	16.9±0.8%	<0.001
Smoking history				<0.001
Never	59.7±1.6%	54.8±3.5%	55.9±1.7%	
Former	22.0±1.3%	28.0±2.5%	29.1±1.4%	
Current	18.3±1.5%	17.2±2.3%	15.0±1.2%	
Kidney disease	2.5±0.3%	3.5±0.8%	3.2±0.5%	<0.001
Liver disease	0%	0%	0%	>0.99
ALT (U/L)	18.7±0.4	19.8±0.7	26.1±0.5	<0.001
AST (U/L)	20.5±0.3	20.3±0.5	22.5±0.3	<0.001
Physical activity (MET-minutes/week)	5255±183	4557±350	4298±202	<0.001
Sedentary time (minutes/day)	341±7	328±10	368±6	<0.001
Sleep time (hours/night)	7.8±<0.1	7.7±<0.1	7.7±<0.1	0.009
Alcohol intake (drinks/week)	3.0±0.1	4.0±0.7	3.5±0.2	0.002
Heavy alcohol intake***	5.3±0.6%	7.3±2.1%	8.3±0.6%	0.02

**Figure 2:**
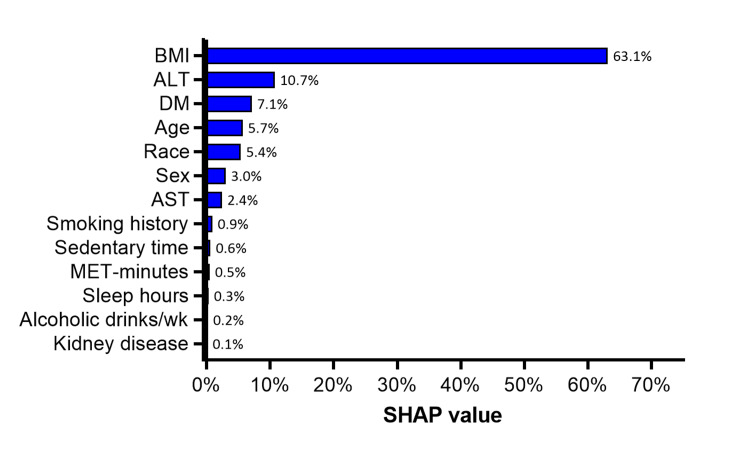
SHAP values indicating the relative importance of covariates in linear regression model to predict liver CAP scores SHAP values are reported on a 0-100% scale. ALT: Alanine aminotransferase; AST: aspartate aminotransferase; CAP: controlled attenuation parameter; DM: diabetes mellitus; MET: metabolic equivalent of task; SHAP: Shapley Additive Explanations.

The relative importance of each covariate in predicting liver CAP was evaluated using SHAP values, with the highest importance given to BMI (63.1%), followed by ALT (10.7%), diabetes mellitus (7.1%), age (5.7%), and race (5.4%). The relative importance of each remaining covariate in the model was less than 5% (Figure 2).

The population prevalence of significant fibrosis was 11.4% (8.3% moderate/severe fibrosis; 3.1% cirrhosis). Comparing individuals with no/mild fibrosis, moderate/severe fibrosis, or cirrhosis, statistically significant differences were noted among all measured covariates except sleep quantity and alcohol consumption (Table 3).

**Table 3:** Association of subject characteristics with liver fibrosis classification assessed by VCTE* *Values are mean ± standard error or percent ± standard error unless otherwise specified. **Population of adults aged 21 years and older with no history of diagnosed liver conditions. ***Defined as ≥15 drinks per week in men and ≥8 drinks per week in women. †P<.05 considered statistically significant. ALT=alanine aminotransferase; AST=aspartate aminotransferase; MET=metabolic equivalent of task; VCTE: vibration-controlled transient elastography.

Variable	No/mild fibrosis (<7.65 kPa)	Moderate/severe fibrosis (7.65-13.00 kPa)	Cirrhosis (>13.00 kPa)	P-value†
Population count**	187,200,000	17,500,000	6,600,000	
Population percentage**	88.6%	8.3%	3.1%	
Age, yr	47.8±0.5	52.4±0.9	52.6±1.1	<0.001
Male sex	47.9±0.8%	54.5±2.6%	61.2±4.5%	<0.001
Race				<0.001
White	62.6±2.4%	63.9±3.4%	70.5±6.4%	
Black	11.6±1.4%	12.8±2.3%	7.9±2.1%	
Asian	6.3±0.9%	3.6±1.2%	3.8±1.1%	
Mexican American	8.1±1.1%	8.6±1.7%	7.3±1.9%	
Other Hispanic	7.5±0.7%	7.1±1.4%	4.9±1.6%	
Other race / multi-race	4.0±0.4%	3.9±1.0%	5.7±2.9%	
Body mass index kg/m^2^	28.8±0.2	35.1±0.6	40.0±1.0	<0.001
Diabetes mellitus	8.6±0.4%	23.3±1.7%	29.9±4.9%	<0.001
Smoking history				<0.001
Never	58.1±1.3%	53.5±3.7%	48.3±4.5%	
Former	25.2±0.9%	30.9±3.6%	35.8±3.8%	
Current	16.7±1.2%	15.6±2.0%	15.8±3.4%	
Kidney disease	2.8±0.3%	3.8±0.7%	4.5±1.5%	<0.001
Liver disease	0%	0%	0%	>0.99
ALT U/L	21.2±0.3	28.5±1.0	35.8±3.3	<0.001
AST U/L	20.7±0.2	24.8±0.9	32.6±3.0	<0.001
Physical activity MET-minutes/week	4853±167	3951±408	3445±573	0.01
Sedentary time minutes/day	348±5	381±13	386±14	0.01
Sleep time hours/night	7.7±<0.1	7.7±0.1	7.6±0.1	0.65
Alcohol intake drinks/week	3.4±0.1	2.7±0.3	3.7±0.9	0.14
Heavy alcohol intake***	6.8±0.5%	5.9±1.3%	11.5±3.7%	0.18

**Figure 3:**
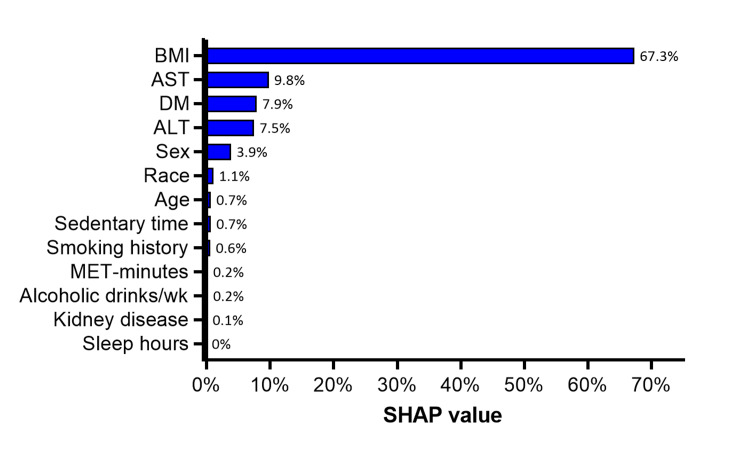
SHAP values indicating the relative importance of covariates in linear regression model to predict liver LSM scores SHAP values are reported on a 0-100% scale. ALT: Alanine aminotransferase; AST: aspartate aminotransferase; DM: diabetes mellitus; LSM: liver stiffness measurement; MET: metabolic equivalent of task; SHAP: Shapley Additive Explanations.

The highest relative importance in predicting LSM was given to BMI (67.3%), followed by AST (9.8%), diabetes mellitus (7.9%), ALT (7.5%), and the remaining variables (<5%) (Figure 3).

**Figure 4:**
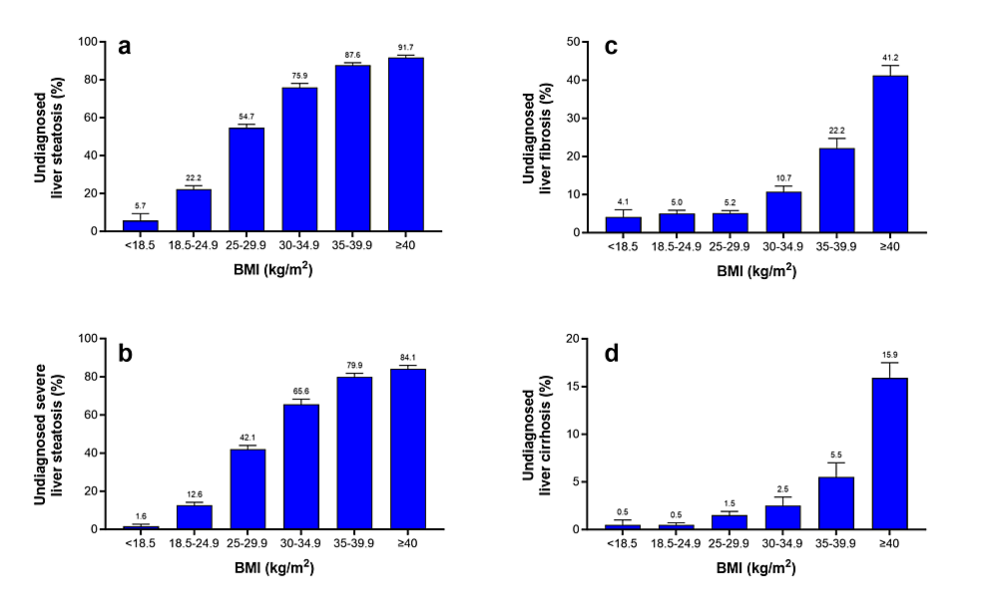
Prevalence of: a) undiagnosed liver steatosis (mild or severe), b) severe liver steatosis, c) significant liver fibrosis (moderate/severe fibrosis or cirrhosis), and d) liver cirrhosis, by BMI category in US adults Values are percentage and standard error. BMI: Body mass index.

The association of the BMI category with the risk of liver steatosis and fibrosis is provided in Figure 4. Steatosis risk linearly increased with increasing BMI, while fibrosis risk remained stable for BMI under 30 kg/m^2^ but exponentially increased with higher BMI. These associations with BMI remained constant when assessing only severe steatosis and cirrhosis, respectively.

Using BMI alone to identify liver steatosis (mild or advanced) resulted in an AUROC was 78.4% (standard error [SE]=0.5%; p<0.01) with maximum discriminatory power at a cutoff of 28.2 kg/m^2^. Similar results were obtained for predicting advanced steatosis only, with an AUROC of 77.3% (SE=0.5%; P<0.001) and a 28.7 kg/m^2^ cutoff. The AUROC for classification of significant fibrosis (moderate/severe fibrosis or cirrhosis) using BMI was 71.8% (SE=1.0%; P<0.001), with a cutoff of 31.7 kg/m^2^, and the AUROC for classification of cirrhosis only was 75.3% (SE=1.7%; P<0.001), with a cutoff of 33.0 kg/m^2^.

## Discussion

Our study revealed that among US adults aged 21 and older without a history of liver disease, VCTE identified liver steatosis and/or significant fibrosis in 59.0% of individuals, with liver steatosis prevalence of 57.2% and significant fibrosis prevalence of 11.4%. BMI emerged as the primary determinant of both measures, with a limited independent contribution from the other covariates such as diabetes mellitus, liver enzymes, age, and alcohol consumption. Although previous studies have reported associations between higher BMI and higher liver steatosis and fibrosis scores [21-23], our study extends these findings in two important ways. First, we limited potential confounding by excluding adults with a history of liver conditions. Second, we used a machine learning approach to identify the relative importance of liver steatosis and fibrosis determinants. These findings indicate that undiagnosed chronic liver disease is highly prevalent in the US adult population, particularly among obese individuals. This is concerning as healthcare providers often prioritize diabetes mellitus diagnosis or liver enzymes over obesity when determining candidates for liver disease screening [4], potentially leaving many at-risk individuals without appropriate medical care. These results suggest that efforts to increase liver disease awareness among patients and providers and to reconsider current BMI thresholds for screening eligibility may be warranted. Such actions could prompt diagnostic testing in patients who might otherwise have been overlooked for liver disease screening, enabling surveillance and treatment earlier in the disease process.

Obesity disrupts hepatic adipokine and hormone release, promoting steatosis, inflammation, and fibrosis. Triglyceride accumulation in hepatocytes comprising at least 5% of liver parenchyma leads to NAFLD. Other proposed mechanisms include alterations in the gut microbiota and dysregulation of intestinal hormones associated with obesity and NAFLD [24,25]. Recent proposals have suggested a reclassification of NAFLD to metabolic-associated fatty liver disease (MAFLD) to highlight the association between liver steatosis and metabolic dysregulation. The proposed criteria for a positive MAFLD diagnosis are based on histological, imaging, or blood biomarker evidence of hepatic steatosis, along with the presence of obesity, type II diabetes mellitus, or metabolic dysregulation. The results of the current study indicate that obesity may be a more significant contributor to liver dysfunction than other metabolic disorders such as diabetes mellitus, as liver steatosis was present in three out of four obese individuals.

The finding that 59.0% of US adults with no prior history of liver disease have liver steatosis or significant fibrosis carries significant public health implications given the strong association of liver steatosis [26] and fibrosis [27] severity with mortality. While the American Association for the Study of Liver Disease recommends routine screening of NAFLD in adults with medically complicated obesity (BMI >40 kg/m^2^, or BMI >35 kg/m^2^ with obesity-related complications) [28], the mean BMI in those with advanced steatosis was only 33 kg/m^2^ in the current study, and 73% of obese adults (BMI >30 kg/m^2^) had advanced steatosis. This suggests that routine liver disease screening based on lower BMI thresholds may be warranted and could help to identify the approximately 100 million US adults with advanced liver steatosis or cirrhosis. Since approximately 40% of individuals with NAFLD progress to cirrhosis within eight years, and eight-year mortality rates are 13% with NAFLD and 31% with NAFLD cirrhosis [7], the potential public health benefits of early detection efforts are considerable and may include reduced disease progression, improved quality of life, and lower healthcare costs. However, the potential drawbacks of this approach must also be recognized. Implementing lower BMI thresholds could considerably increase the number of individuals identified as at risk, potentially placing a significant strain on healthcare resources. Furthermore, although BMI is an important predictor of liver steatosis and fibrosis, its independent predictive power is limited, potentially leading to inaccuracies in risk assessment. These complexities highlight the need for future studies to explore the clinical and economic tradeoffs of applying lower BMI thresholds for liver disease screening.

This population-based study has several important strengths. First, the results are generalizable to the US population of adults without a previous diagnosis of liver disease, contrasting with much prior work on correlates of VCTE measures that included individuals with prior liver disease [21,22] or smaller samples with limited generalizability [29,30]. Second, utilizing NHANES allowed for the evaluation of population-based health characteristics across many different sociodemographic characteristics that could not be accurately modeled in smaller studies. Third, we determined the relative importance of covariates in predicting VCTE parameters using SHAP values, which allowed practical interpretation of regression model outputs. A final strength of this study was the inclusion of heavy drinkers, comprising 6.9% of the sample. This allowed for the simultaneous assessment of the relative importance of alcohol intake alongside BMI and other variables. Despite including heavy drinkers, the study found that alcohol intake had minimal contribution compared to BMI in determining liver steatosis and significant fibrosis.

Despite these strengths, this study had several limitations. First, the cross-sectional study design prevents determining causal relationships between the observed associations and VCTE parameters. Second, the self-reported components of the NHANES data may introduce inaccuracies due to recall and response bias. Third, model accuracy was higher for detecting liver steatosis than fibrosis. Factors such as medication use, long-term exposure to environmental toxins, and social determinants, which were not measured in this study, may influence liver fibrosis detection. Fourth, the reliability of VCTE measures may be compromised in morbidly obese individuals due to increased skin-to-liver capsule distance, although using a larger probe can largely mitigate this issue. Fifth, while we utilized results of previous meta-analyses to define liver steatosis and fibrosis categories [14,15], no universally accepted categorical thresholds exist, which may limit interpretation when comparing results to previous VCTE studies. Finally, the study was purposely designed to broadly identify high-risk undiagnosed individuals irrespective of specific liver disease subtypes such as NAFLD. Although the current BMI threshold recommendations for liver disease screening apply to NAFLD [28], 93.4% of the study sample were not heavy drinkers, and, therefore, the recommendation to consider lower BMI screening limits likely remains valid across the population.

## Conclusions

Undiagnosed chronic liver disease based on VCTE findings is highly prevalent among US adults, particularly in individuals with obesity. Demographics, diabetes mellitus, liver enzyme concentrations, and lifestyle factors provided little additional predictive value. The high prevalence of undiagnosed liver disease revealed in this nationally representative study indicates that current practices to detect chronic liver disease early may be inadequate. Efforts to increase awareness about liver disease among patients and providers and to reconsider existing BMI thresholds for liver disease screening may be warranted. Integration of liver disease screening into routine primary care, particularly for overweight and obese adults, could promote earlier diagnosis and treatment to reduce population morbidity and mortality.
